# The Use of Mutational Signatures to Decipher the Inter-Relationship of Reactive Oxygen Species with Homologous Recombination and Non-Homologous End-Joining Deficiencies as Well as Their Effects on APOBEC Mutagenesis in Breast Cancer

**DOI:** 10.3390/cancers17101627

**Published:** 2025-05-12

**Authors:** Amir Farmanbar, Robert Kneller, Sanaz Firouzi

**Affiliations:** 1 Department of Computational Biology and Medical Sciences, Graduate School of Frontier Sciences, The University of Tokyo, Tokyo 108-8639, Japan; 2 Research Center for Advanced Science and Technology, University of Tokyo, Tokyo 153-8904, Japan

**Keywords:** breast cancer, DNA damage, mutational signature, BRCA1, BRCA2, AID/APOBEC, activation-induced deaminase/apolipoprotein B mRNA editing enzyme catalytic polypeptide-like, MSI, microsatellite instability, HRD, homologous recombination deficiency, ROS, reactive oxygen species, NHEJ, non-homologous end joining, TMB, tumor mutational burden

## Abstract

**Simple Summary:**

The presence of certain DNA damage/repair errors together can cause cancer cells to die, offering hope for new treatments. Mutational signatures show how DNA damage/repair errors happened by looking at the types and amounts of mutations. We studied mutational signatures that occur together or separately, called mutational signature interactions, to find which combinations of DNA repair errors help cancer cells thrive and which ones harm them. We analyzed mutational signatures and their interactions in 1014 breast cancer genomes. HRd and NHEJd were found together with APOBEC, while they did not overlap with the ROS mutational signature. Mutational signature interactions were distinct in various breast cancer types. Non-HRd TNBC tumors showed a unique ROS signature, while HRd TNBC tumors did not have an ROS signature. These analyses show that mutational signatures and their interactions could help identify promising synthetic lethal targets in DNA repair systems, improve how patients are grouped and point to potential treatments.

**Abstract:**

**Background:** Defective DNA repair systems result in the accumulation of mutations, loss of genomic integrity, and eventually cancer. Following initial malignant transformation due to specific DNA damage and defective DNA repair, cancer cells become reliant upon other DNA repair pathways for their survival. The co-occurrence of specific repair deficiencies brings catastrophic outcomes such as cell death for cancer cells and thus holds promise as a potential therapeutic strategy. Exploring the co-occurrence and mutual exclusivity of mutational signatures provides valuable knowledge regarding combinations of defective repair pathways that are cooperative and confer selective advantage to cancer cells and those that are detrimental and cannot be tolerated by them. **Methods:** Taking advantage of mutational signature profiling, we analyzed whole-genome sequences of 1014 breast cancers to reveal the underlying mutational processes and their interrelationships. **Results:** We found an inverse relationship between deficiencies of homologous recombination (HRd) and non-homologous end joining (NHEJd) with reactive oxygen species (ROS). Moreover, HRd and NHEJd co-occurred with APOBEC but were mutually exclusive with mismatch repair deficiency (MMRd) and ROS. Our analysis revealed that SBS8 and SBS39 signatures of currently unknown etiology correlate with NHEJd. ID1 and ID2 signatures co-occur with ROS and have mutual exclusivity with HRd, SBS8, SBS39 and NHEJd. The ID4 signature, with currently unknown etiology, has mutual exclusivity with HRd and NHEJd and co-occurred with ROS. On the other hand, the ID15 signature, with currently unknown etiology, co-occurred with SBS8, SBS39, HRd, NHEJd and DBS2, while having an inverse relationship with MMRd and ROS. Comparing the mutational signatures of HRd and non-HRd TNBC genomes reveals the unique presence of ROS signatures in non-HRd tumors and the lack of ROS signature in HRd tumors. **Conclusion:** Taken together, these analyses indicate the possible application of mutation signatures and their interactions in advancing patient stratification and suggest appropriate therapies targeting the make-up of individual tumors’ mutational processes. Ultimately, this information provides the opportunity to discover promising synthetic lethal candidates targeting DNA repair systems.

## 1. Introduction

The integrity of the genome is maintained through successful DNA replication and faithful DNA repair, while it is jeopardized by endogenous and exogenous sources of DNA damage well as impaired DNA repair pathways [1]. Depending on the nature of DNA damage, specific pathways become activated to identify damaged regions and repair them, collectively defined as the DNA damage response (DDR). Impaired DDR threatens genome stability and incites cancer development [2]. Mutational processes leading to accumulated mutations with unique characteristic patterns known as mutational signatures provide the means to detect and quantify contributions of DNA damage/repair pathways in tumor mutation burden (TMB) [3,4,5]. Employing mutational signatures as an integrative approach for classifying patients may turn genomic instability into an advantage for selecting therapeutic targeting.

DNA double-strand breaks (DSBs) are the most deleterious type of damage, and they are repaired by non-homologous end-joining (NHEJ) and homologous recombination (HR) pathways [6,7]. Unlike HR pathways, because NHEJ does not require homologous DNA templates, NHEJ is an error-prone repair process. Inhibition of NHEJ pathways has been shown to increase susceptibility of tumors to radio- and chemotherapy, suggesting components of NHEJ pathways are possible candidates for targeted therapy [8]. Platinum therapies and poly-ADP ribose polymerase (PARP) inhibitors show favorable outcomes in treating tumors with HR deficiency (HRd), rendering reliable detection of HRd status highly critical for patient selection in different cancer types [9,10]. While the significant role of HR genes including BRCA1/2 in breast tumorigenesis has been clearly demonstrated, solid evidence to connect defective NHEJ genes with breast cancer development remains to be found. A deeper understanding of HRd and NHEJ deficiency (NHEJd) and their actionability in breast cancer facilitates development of DNA repair-mediated targeted therapies.

Mismatch repair (MMR) and base excision repair (BER) are two main pathways responsible for genome maintenance via repairing mispaired bases or DNA single-strand break damages [11,12,13]. MMR deficiency (MMRd) of tumors results in increased antigenicity and better response to immunotherapeutic approaches such as immune checkpoint inhibitors. Notably, mutual exclusivity of MMRd and HRd signatures has been shown in different malignancies including gynecological, stomach and colorectal cancers [14,15]. Possible interaction of MMRd and BERd with other mutational signatures and their potential role in prognosis remains to be elucidated in breast cancer. Recent studies propose that relationships between BER and non-HR DNA repair pathways may hold potential for synthetic lethality [16]. Indeed, MSH1 and MSH2 deficiencies are synthetically lethal with the inhibition of POLG and POLB, as well as the BER constituent of DNA polymerases, respectively [17].

While the natural editing function of the apolipoprotein-B mRNA-editing catalytic polypeptidelike 3 (APOBEC3) family of cytidine deaminases mediates the restriction of retroviruses, endogenous retro-elements and DNA viruses, APOBEC3 dysregulation severely affects genome integrity [18,19]. APOBEC3-dependent mutagenesis is a major source of genomic instability in multiple human cancer types, such as urothelial, cervical and breast tumors [20,21]. Co-occurrence of APOBEC mutational signatures (SBS2, 13) with HRd has been documented in different cancer types such as gynecological [14], colorectal and stomach [15] malignancies, suggesting possible cooperative functions. Clarifying their prevalence and links to DNA repair pathways may facilitate finding suitable targeted therapeutic intervention in breast cancer.

So far, SBS18 is the only signature with a unique pattern of mutations attributed to reactive oxygen species (ROS) etiology [22]. Tumor samples with SBS18 have been reported in several different cancer types [4], and it is predominant in neuroblastoma [23,24]. The mutation pattern of SBS18 is dominated by C > A transversion, indicating ROS-induced 8-oxoguanine damage formation [5,25]. ROS are multifaceted highly reactive, oxygen-containing molecules with two major paradoxical roles of promoting pro-tumorigenic and anti-tumorigenic signaling. Although moderate levels of ROS are required for several cellular functions, excessive levels act as primary sources of genome instability and are known as emerging hallmarks of cancer. Elevated antioxidant capacity of cancer cells confers upon them the ability to maintain ROS hemostasis and avoid cell death. Therefore, altering the redox environment of cancer cells makes them susceptible to ROS manipulation therapies [26,27]. However, it is still a major challenge to translate ROS knowledge from bench to beside and effectively target ROS for cancer therapy.

The mutational signatures belong to a class of emerging cancer biomarkers which connect somatic DNA mutations to exogenous and endogenous DNA damage and repair defects. Profiling mutational signatures assists in determining patients who may benefit from certain therapies targeting their mutational make-up. The application of mutational signatures in therapeutic decision-making is an active area of research. While targeted therapy based on signatures such as HRd pioneers in the field, others such as APOBEC signatures are under active development. Recent studies documented the association of APOBEC signatures with sensitivity to inhibition of ataxia telangiectasia and Rad3-related kinase (ATR) [28,29,30].

The high degree of heterogeneity is the major hurdle for cancer treatment in different cancer types [31,32,33,34]. Heterogeneity hampers accurate patient categorization, particularly in breast cancer cases, whose classification has proven to be central for proper patient management, follow-up and therapeutic selection [33,35]. Indeed, a high degree of heterogeneity in breast tumors, even among those belonging to the same subtype, makes predicting the therapeutic response a major challenge [36,37], underscoring the need to develop precise classifications to direct appropriate targeted therapies for each individual tumor. One of the major clinically challenging types of breast cancer, known as triple-negative breast cancer (TNBC), is negative for hormone receptors (HRs), i.e., estrogen receptors (ER-), progesterone receptors (PR-), and excess human epidermal growth factor receptor 2 (HER2) proteins. Unlike other types of breast cancer such as ER+ or PR+ breast cancer, due to a lack of effective targeted therapies, there are limited treatment options for TNBC cases, as well as refractory breast tumors, which are resistant to currently available therapies [38]. Emerging evidence strongly supports enhancing the effectiveness of therapy by targeting DNA damage/repair fundamentally [39,40]. Towards this end, herein, we aimed to categorize breast tumors more specifically using defects in the DNA damage/repair pathways.

## 2. Materials and Methods

Mutation data source: We employed Simple Somatic Mutation (SSM) files for 1014 whole-genome sequencing (WGS) of breast cancer samples from the data portal of the International Cancer Genome Consortium (ICGC, https://dcc.icgc.org/projects, accessed on 7 May 2025) (*n* = 777) [41] and (*n* = 237) [42]. In brief, mutations including single-base substitutions (SBSs), double-base substitutions (DBSs) and small insertions/deletions (IDs) were extracted from SSM files, converted to mutation matrices and utilized for subsequent analyses.

Mutational signature analysis: We performed a non-negative matrix factorization (NMF)-based de novo mutational signature analysis using GRCh37 and the Catalogue of Somatic Mutations in Cancer (COSMIC, V3.1) (http://cancer.sanger.ac.uk/cosmic/signatures), by (https://github.com/alexandrovlab, accessed on 7 May 2025) [43,44].

The first and second dominant signatures were identified and visualized based on their contribution values in each tumor. The R packages “car”, “ComplexHeatmap”, “circlize”, “ternary”, “vioplot” and “ggplot” were used for visualization [45,46].

Statistical analyses: *p* values were calculated using the Mann–Whitney U test, Kolmogorov–Smirnov (K.S.) test, Kruskal–Wallis rank sum (K.W.) test, hypergeometric test, or Fisher’s exact test. Hypergeometric test for examining statistical independence was used to determine mutually exclusive/co-occurring pairs. All analyses were conducted in the R statistical environment (R version v4.3.1 http://www.r-project.org/). All reported *p* values were two-tailed; ≤0.05 was considered significant.

## 3. Results

### 3.1. Mutational Signatures of Breast Tumors Genomes Reveal Mutual Exclusivity of HRd and NHEJd with ROS and MMRd as Well as Their Co-Occurrence with APOBEC

**Figure 1:**
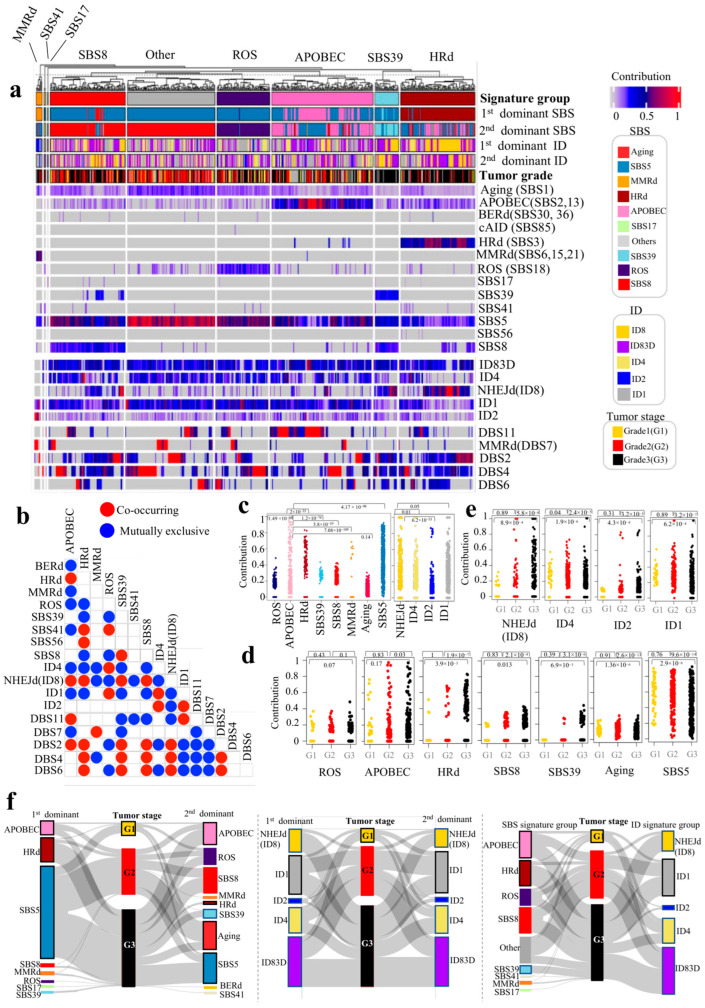
Mutational signature analysis of whole-genome sequencing (WGS) breast tumors. We analyzed SBSs, IDs, and DBSs in 569 whole genomes of breast tumors. (**a**) Non-negative matrix factorization (NMF)-based de novo mutational signatures of tumors visualized by a heatmap divided based on distinct signature status. The signature group, the first and second dominant SBS and ID signatures and the tumor grades are annotated at the top. The contribution values of each signature are shown by a color scale. Color codes representing each dominant mutational signature are shown. For mutational signatures with known etiology, both signature and etiology are indicated. (**b**) Interaction of signatures with each other measured by hypergeometric test: red represents co-occurrence and blue represents mutual exclusivity. (**c**) Contribution values of SBS and ID mutational signatures. (**d**) Comparing contribution values of SBS signatures in different tumor stages. (**e**) Comparing contribution values of ID signatures in different tumor stages. (**f**) Dominant signature in genomes of different grades of tumors: left—the first and second dominant SBS mutational signatures; middle—the first and second dominant ID mutational signatures; right—signature groups of different grades of tumors. Dominant signatures were based on the contribution value of detected mutational signatures. As denoted in the color gradient guide above, the exposure of each signature, i.e., contribution, has values between 0 and 1. We allocated gray to a value of zero and red to a value of 1, then made a gradient of colors with different shades of blue and red corresponding to each contribution value. Mutational signature groups are assigned based on the first and second dominant signature of each sample. The Others signature group is assigned when the first and/or second dominant signature of a sample is SBS1 (aging) and/or SBS5.

We profiled mutational signatures and their interactions in the first dataset of 569 cases with different types of breast cancer (Supplementary Figure S1). In breast tumor genomes, the presence and contribution of detected signatures enabled us to distinguish tumor groups with distinct patterns of mutational signatures, five of which with higher prevalence were dominated by APOBEC (SBS2, 13), HRd (SBS3), SBS8, ROS (SBS18) and SBS39 (Figure 1). As described below, Figure 1 and Figures S2 and S3 show detected mutational signatures, their interactions, contributions and dominant signatures.

APOBEC SBS signatures (SBS2, 13) showed the highest prevalence and were detected in (373 out of 569) 66.43% of tumors. APOBEC SBS signatures were the first dominant signature in (65 out of 569) 11.42% and the second dominant signature in (101 out of 569) 17.75% of tumors. The average contribution value of APOBEC signatures was 0.11 (range: 0.04–0.98). APOBEC signatures were mutually exclusive with MMRd, ROS, BERd (SBS30, 36), SBS39, SBS41, ID4, ID2 and ID1, while they co-occurred with HRd, DBS2 and DBS11.

An HRd (SBS3) signature was detected in (109 out of 569) 19.16% of tumors. HRd was the first dominant signature in (96 out of 569) 16.87% and the second dominant signature in (13 out of 569) 2.28% of tumors. The average contribution value of HRd was 0.5 (range: 0.23–0.83). HRd was mutually exclusive with ROS, SBS39, SBS8, ID1 and ID4, while it co-occurred with APOBEC, SBS41, SBS56, NHEJd (ID8), DBS2, DBS4 and DBS6.

We detected MMRd SBS signatures, including SBS26 and SBS44, in (9 out of 569) 1.58% of tumors. MMRd was the first dominant signature in (8 out of 569) 1.41% and the second dominant signature in (1 out of 569) 0.18% of tumors. MMRd had the highest TMB compared to the rest of signatures. The average contribution value of MMRd signatures was 0.54 (range: 0.25–0.69). SBS MMRd signatures were mutually exclusive with APOBEC, ID4, NHEJd (ID8) and DBS4, while they co-occurred with DBS7 (MMRd).

An SBS8 signature was detected in (176 out of 567) 30.93% of tumors. SBS8 was the first dominant signature in (9 out of 569) 1.58% and the second dominant signature in (103 out of 569) 18.1% of tumors. The average contribution value of SBS8 was 0.24 (range: 0.15–0.43). SBS8 was mutually exclusive with HRd, ROS, ID1, ID4 and DBS11, while it co-occurred with SBS39, NHEJd (ID8), DBS2, DBS4 and DBS6.

An SBS39 signature was detected in (52 out of 569) 9.14% of tumors. SBS39 was the first dominant signature in (6 out of 569) 1.05% and the second dominant signature in (28 out of 569) 4.92% of tumors. The average contribution value of SBS39 was 0.28 (range: 0.17–0.43). SBS39 was mutually exclusive with HRd, ROS, ID1, ID4, DBS7 and DBS11, while it co-occurred with SBS8, NHEJd (ID8), DBS2, DBS4 and DBS6.

An ROS (SBS18) signature was detected in (135 out of 569) 23.73% of tumors. ROS was the first dominant signature in (1 out of 569) 0.18% and the second dominant signature in (70 out of 569) 12.3% of tumors. The average contribution value of ROS was 0.19 (range: 0.1–0.48). ROS was mutually exclusive with APOBEC, HRd, SBS8, SBS39, NHEJd (ID8), DBS2 and DBS6, while it co-occurred with SBS41, ID1 and ID4.

A BERd (SBS30, 36) signature was detected in (9 out of 569) 1.58% of tumors. BERd was the first dominant signature in (0 out of 569) 0% and the second dominant signature in (1 out of 569) 0.18% of tumors. The average contribution value of BERd was 0.14 (range: 0.08–0.35). BERd was mutually exclusive with APOBEC.

An SBS17 signature was detected in (9 out of 569) 1.58% of tumors. SBS17 was the first dominant signature in only one case and the second dominant signature in none of the tumors. The average contribution value of SBS17 was 0.14 (range: 0.04–0.58). SBS17 did not show any interaction with other signatures.

An SBS41 signature was detected in (20 out of 569) 3.51% of tumors. SBS41 was the second dominant signature in two cases (0.35%). The average contribution value of SBS41 was 0.15 (range: 0.11–0.22). SBS41 was mutually exclusive with APOBEC, HRd, NHEJd (ID8) and DBS11, while it co-occurred HRd and ROS.

An SBS56 signature was detected in (4 out of 569) 0.7% of tumors. SBS56 was not dominant in any case. The average contribution value of SBS56 was 0.07 (range: 0.06–0.09). SBS56 co-occurred with HRd. In addition, only one case showed cAID (SBS85), with a contribution value of 0.14, and did not show any interaction with other signatures.

An NHEJd (ID8) signature was detected in (222 out of 569) 39.02% of tumors. NHEJd (ID8) was the first dominant signature in (82 out of 569) 14.4% and the second dominant signature in (69 out of 569) 12.13% of tumors. The average contribution value of NHEJd (ID8) was 0.37 (range: 0.10–1). NHEJd (ID8) was mutually exclusive with ROS, SBS MMRd, SBS41, ID1, ID2 and DBS11, while it co-occurred with HRd, APOBEC, SBS39, SBS8, DBS2, DBS4 and DBS6.

An MMRd (DBS7) signature was detected in (62 out of 569) 10.9% of tumors. The average contribution value of MMRd (DBS7) was 0.74 (range: 0.2–1). MMRd (DBS7) was mutually exclusive with APOBEC, SBS39, DBS2, DBS4 and DBS6, while it co-occurred with MMRd SBS signatures.

The contribution values of each mutational signature in both SBS and ID showed significantly different patterns (*p* value < 2.2 × 10^−16^, K.W. test) (Figure 1c). Similarly, significance differences were observed in pair-wise comparisons. For instance, APOBEC signatures were significantly different from any other SBS signatures, and the NHEJd (ID8) signature was significantly different from any other ID signatures (Figure 1c).

### 3.2. Different Tumor Stages of Breast Tumors Significantly Differ in Their Mutational Signatures’ Profiles and Distribution

Grading in breast cancer is an important factor in predicting how quickly the cancer might grow and spread, which mainly assists in clinical settings to predict outcome and select the appropriate therapies. Grade1 (G1) tumors are low-grade, slow-growing and well-differentiated; Grade2 (G2) tumors are intermediate-grade and moderately differentiated; and Grade3 (G3) tumors are high-grade, fast-growing and poorly differentiated.

We stratified the tumor stages of the breast tumors based on their mutational signature profiles, first and second dominant signature and signature group. Enrichment of signatures in each tumor stage was significantly different (first dominant signatures: 1.5 × 10^−5^, second dominant signatures: 4.54 × 10^−10^, SBS signature groups: 3.6 × 10^−14^, ID signature groups: 1.17 × 10^−6^ [*p* values; Fisher’s exact test] (Figure 1d–f).

Grade1 tumors consisted of cases with first dominant signatures of SBS5 (80%), APOBEC (12%) and HRd (8%). Grade2 tumors consisted of cases with first dominant signatures of SBS5 (81%), APOBEC (10%), HRd (7%), SBS8 (1%) and MMRd (1%). Grade3 tumors consisted of cases with first dominant signatures of SBS5 (59%), HRd (26%), APOBEC (8%), SBS8 (3%), MMRd (2%) and SBS39 (2%) (Figure 1f left).

Grade1 tumors consisted of cases with second dominant signatures of Aging (28%), ROS (25%), SBS5 (18%), SBS8 (18%) and APOBEC (12%). Grade2 tumors consisted of cases with second dominant signatures of Aging (38%), SBS8 (21%), SBS5 (16%), APOBEC (12%), SBS39 (1%) and MMRd (1%). Grade3 tumors consisted of cases with second dominant signatures of SBS5 (27%), APOBEC (20%), SBS8 (19%), Aging (11%), SBS39 (9%), SBS8 (3%) and HRd (3%) (Figure 1f left).

Grade1 tumors consisted of cases with SBS signature groups of Other (28%), APOBEC (25%), ROS (25%), SBS8 (15%) and HRd (8%). Grade2 tumors consisted of cases with SBS signature groups of Other (37%), APOBEC (16%), SBS8 (21%), ROS (12%), SBS41 (1%) and MMRd (1%). Grade3 tumors consisted of cases with SBS signature groups of HRd (27%), APOBEC (20%), SBS8 (19%), Other (10%), ROS (12%), SBS39 (10%) and MMRd (2%) (Figure 1f right).

Grade1 tumors consisted of cases with ID signature groups of ID83D (42%), ID4 (32%) and ID1 (25%). Grade2 tumors consisted of cases with ID signature groups of ID83D (36%), ID1 (31%), ID4 (24%), NHEJd (ID8) (7%) and ID2 (2%). Grade3 tumors consisted of cases with ID signature groups of ID83D (34%), ID1 (26%), NHEJd (ID8) (23%), ID4 (14%) and ID2 (3%) (Figure 1f middle).

HRd, SBS8, SBS39 and NHEJd signatures are significantly enriched in Grade3 tumors, and these signatures tend to show higher contribution values in Grade3 tumors compared to lower grades. In contrast, APOBEC and ROS showed similar enrichment in Grade2 and Garde3 tumors with no significant difference in contribution values (Figure 1d–f).

**Figure 2:**
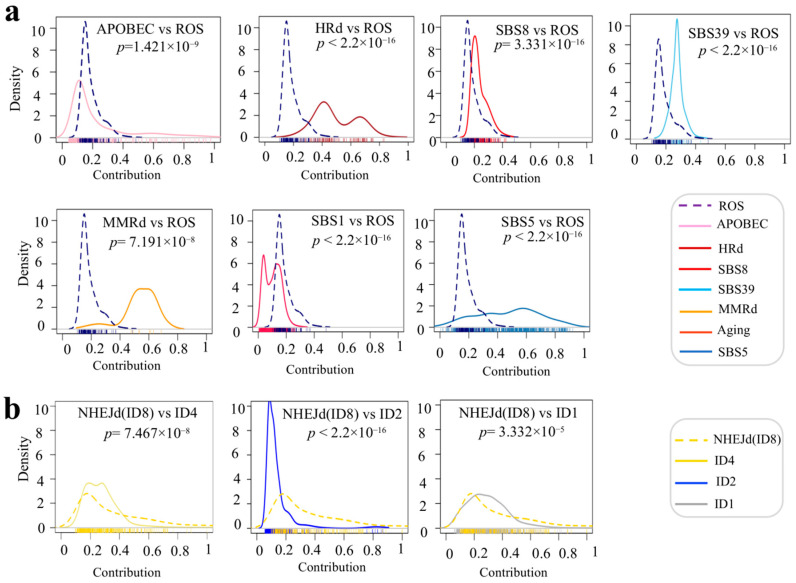
Kernel density comparing distribution of mutational signatures’ relative contribution in breast tumors. (**a**) Distribution of relative contribution for ROS compared with other SBS mutational signatures in density plots. (**b**) Distribution of relative contribution for NHEJd (ID8) compared with other ID mutational signatures in density plots. The *X* axis represents contribution. Ticks for each contribution value are presented under the distribution curves. The *Y* axis is a kernel density estimate to show the probability density. Only samples with counts more than zero are shown. *p* values were calculated using K.S. test.

We observed significant differences between the distribution shapes of the ROS signature and those of other SBS signatures, as well as between NHEJ (ID8) and other ID signatures in breast tumors, as shown by kernel density plots in Figure 2. Kernel plots further depict the extent and magnitude of difference between signature groups. ROS showed a shift towards lower contribution values with very narrow spread. Large numbers of samples with APOBEC had a tendency towards low contributions, but a subset of them with higher contributions generated a quite long tail in the plot. HRd showed a distribution with two peaks at 0.4 and 0.7. While SBS1 and SBS5 are both aging-related signatures, they displayed significantly different distributions. Most of the samples had SBS1 but with a distribution towards very low contributions. However, samples with SBS5 showed widespread contributions. NHEJd (ID8) showed an asymmetrical distribution with a long tail towards higher contributions. ID2 showed a narrow spread towards low contributions. ID1 showed a relatively normal distribution (Figure 2).

### 3.3. Mutational Signatures of TNBC Genomes Reveal Mutual Exclusivity Between ROS and HRd, SBS8 and 39 Signatures, as Well as Co-Occurrence of APOBEC with HRd and NHEJd

**Figure 3:**
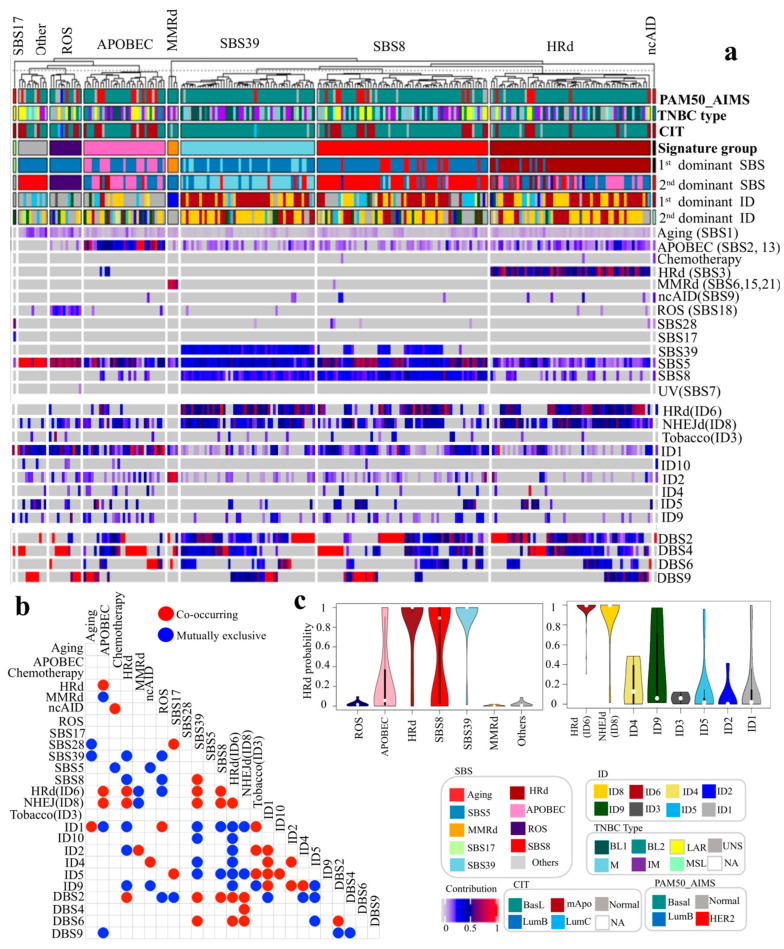
Mutational signature analysis of TNBC whole-genome sequencing (WGS) tumors. We analyzed SBSs, IDs and DBSs in 237 whole genomes of TNBC tumors. (**a**) NMF-based de novo mutational signatures of TNBC tumors visualized by a heatmap divided based on distinct signature status. The signature group, the first and second dominant SBS and ID signatures and tumor staging are annotated at the top. Different tumor staging systems, including PAM50_AIMS, TNBC type and CIT, are shown. The contribution values of each signature are shown by a color scale. Color codes representing each dominant mutational signature are shown. For mutational signatures with known etiology, both signature and etiology are indicated. (**b**) Interaction of signatures with each other measured by hypergeometric test: red represents co-occurrence and blue represents mutual exclusivity. (**c**) Comparing HRd scores of different SBS and ID signatures. Mutational signature groups are assigned based on the first and second dominant signature of each sample. The Others signature group is assigned when the first and/or second dominant signature of a sample is SBS1 (aging) and/or SBS5.

In 237 analyzed TNBC genomes, the presence and contribution of detected signatures enabled us to distinguish tumor groups with distinct patterns of mutational signatures, the five of which with the highest prevalence were dominated by HRd (SBS3), SBS8, SBS39, APOBEC and ROS, respectively (Figure 3). As described below, Figure 3a,b and Supplementary Figure S4 show detected mutational signatures, their interactions, contributions, and dominant signatures.

APOBEC SBS signatures (SBS2, 13) were detected in (155 out of 237) 65% of tumors. APOBEC SBS signatures were the first dominant signature in (16 out of 237) 6.7% and the second dominant signature in (36 out of 237) 15% of tumors. The average contribution value of APOBEC signatures was 0.2 (range: 0.05–1). APOBEC signatures were mutually exclusive with MMRd, ID1 and DBS9, while they co-occurred with HRd, HRd (ID6) and NHEJd (ID8).

An HRd (SBS3) signature was detected in (64 out of 237) 27% of tumors. HRd (SBS3) was the first dominant signature in (56 out of 237) 24% and the second dominant signature in (8 out of 237) 3% of tumors. The average contribution value of HRd (SBS3) was 0.54 (range: 0.31–0.84). HRd (SBS3) was mutually exclusive with SBS39, SBS8, ID1, ID2 and ID9, while it co-occurred with APOBEC, HRd (ID6), NHEJd (ID8) and DBS2.

An SBS8 signature was detected in (144 out of 237) 61% of tumors. SBS8 was the first dominant signature in (13 out of 237) 5.5% and the second dominant signature in (65 out of 237) 27% of tumors. The average contribution value of SBS8 was 0.26 (range: 0.1–0.6). SBS8 was mutually exclusive with ROS, ID1and ID5, while it co-occurred with SBS39, HRd (ID6), NHEJd (ID8) and DBS2.

SBS MMRd signatures were detected in (5 out of 237) 2% of tumors. MMRd was the first dominant signature in four tumors. The average contribution value of MMRd was 0.62 (range: 0.1–0.82). MMRd signatures were mutually exclusive with APOBEC, HRd (ID6) and NHEJd (ID8), while they co-occurred with ID2.

An ROS (SBS18) signature was detected in 20 out of 237 (8%) of tumors. ROS was the second dominant signature in (12 out of 237) 5% of tumors. The average contribution value of ROS was 0.1 (range: 0.09–0.3). ROS was mutually exclusive with SBS39, SBS8, HRd (ID6) and DBS2, while it co-occurred with ID1.

An SBS39 signature was detected in (77 out of 237) 32.5% of tumors. SBS39 was the first dominant signature in (13 out of 237) 5.5% and the second dominant signature in (41 out of 237) 17% of tumors. The average contribution value of SBS39 was 0.29 (range: 0.18–0.41). SBS39 was mutually exclusive with Aging, HRd, ROS, ID1, ID4, ID5 and ID10, while it co-occurred with SBS8, HRd (ID6), NHEJd (ID8), DBS2 and DBS6.

An SBS17 signature was detected in (8 out of 237) 3% of tumors. SBS17 was the first dominant signature in only one case. The average contribution value of SBS17 was 0.15 (range: 0.05–0.6). SBS17 was mutually exclusive with DBS2, while it co-occurred with SBS28 and ID5.

An ncAID (SBS9) signature was detected in (11 out of 237) 5% of tumors. ncAID was the first dominant signature in (1 out of 237) and the second dominant signature in (3 out of 237) 1% of tumors. The average contribution value of ncAID was 0.22 (range: 0.14–0.39). ncAID was mutually exclusive with SBS5 and DBS2, while it co-occurred with chemotherapy and ID4.

We also compared HRd scores in different signature groups. Figure 3c depicts the distribution of HRd scores in each signature group. ROS, MMRd and other (SBS1 (aging) and SBS5) groups showed the lowest HRd scores, with mean values of 0.02, 0.004 and 0.02, respectively. SBS39, HRd, SBS8 and APOBEC, had the highest HRd scores, with mean values of 0.92, 0.84, 0.56 and 0.27, respectively. In ID signature groups, HRd (ID6) and NHEJd (ID8) showed the highest HRd score, with mean values of 0.98 and 0.91, respectively. On the other hand, ID9, ID4, ID1, ID5, ID2 and ID3 showed the lowest HRd scores, with mean values of 0.36, 0.20, 0.16, 0.13, 0.06 and 0.06, respectively. HRd scores of both SBS and ID signature groups were significantly different (*p* values 4.8 × 10^−15^ and <2.2 × 10^−16^, K.W test) (Figure 3c).

### 3.4. Comparing Mutational Signatures of HRd and Non-HRd TNBC Genomes Reveals Unique Presence of ROS Signature in Non-HRd Tumors and Lack of ROS Signature in HRd Tumors

**Figure 4:**
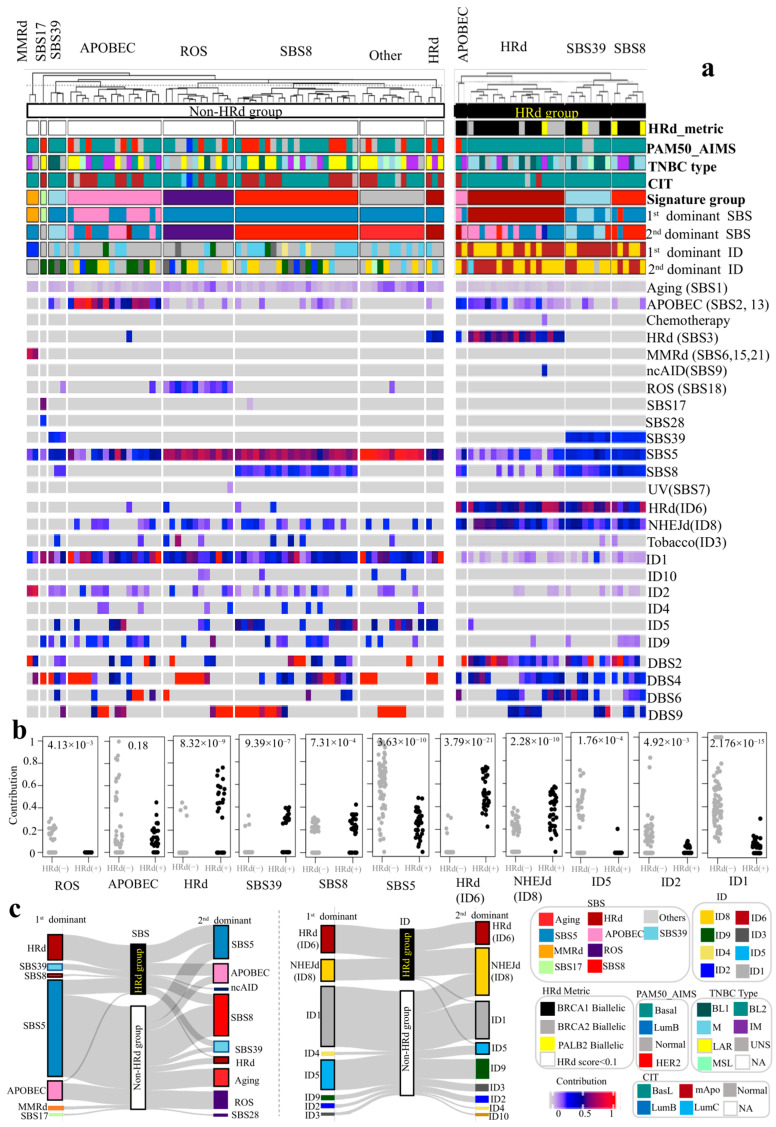
Comparing mutational signatures of TNBC tumors with HRd and non-HRd status. TNBC cases were assigned to two distinct group of HRd and non-HRd. The HRd group contains tumors with bi-allelic mutations of BRCA1/2 or PALB2, while the non-HRd TNBC group contains tumors with an HRd score less than 0.1. (**a**) NMF-based de novo mutational signatures of HRd TNBC tumors (right) and non-HRd TNBC tumors (left) visualized by a heatmap divided based on distinct signature status. The signature group, the first and second dominant SBS and ID signatures and tumor staging are annotated at the top. Different tumor staging systems, including PAM50_AIMS, TNBC type and CIT, are shown. The contribution values of each signature are shown by a color scale. Color codes representing each dominant mutational signature are shown. For mutational signatures with known etiology, both signature and etiology are indicated. (**b**) Comparing contribution values of SBS and ID signatures in HRd and non-HRd TNBC tumors [HRd (+): black dots vs. HRd (−): gray dots]. *p* values were calculated using the Mann–Whitney U test. (**c**) Dominant signature analysis of TNBC tumors with HRd and non-HRd status: left—the first and second dominant SBS mutational signatures; right—the first and second dominant ID mutational signatures. Mutational signature groups are assigned based on the first and second dominant signature of each sample. The Others signature group is assigned when the first and/or second dominant signature of a sample is SBS1 (aging) and/or SBS5.

We divided TNBC cases into those with HRd and without HRd signatures. Non-HRd TNBC tumors have an HRd score less than 0.1. The HRd group contain tumors with bi-allelic mutations of BRCA1/2 or PALB2 (Figure 4).

APOBEC SBS signatures (SBS2, 13) were detected in (23 out of 33) 69.7% of HRd_TNBC tumors. APOBEC SBS signatures were the first dominant signature in (1 out of 33) 3.03% and the second dominant signature in (8 out of 33) 24.24% of these tumors. The average contribution value of APOBEC signatures was 0.17 (range: 0.06–0.45). In contrast, APOBEC SBS signatures (SBS2, 13) were detected in (32 out of 69) 46.38% non-HRd_TNBC tumors. APOBEC SBS signatures were the first dominant signature in (11 out of 69) 15.94% and the second dominant signature in (5 out of 69) 7.25% of these tumors, and the average contribution value of APOBEC signatures was 0.35 (range: 0.05–1). In sum, APOBEC showed higher prevalence but lower contribution value in HRd_TNBC tumors compared to non-HRd_TNBC tumors (Figure 4).

The SBS3 HRd signature was detected in (18 out of 33) 27% of HRd_TNBC tumors. HRd (SBS3) was the first dominant signature in (17 out of 33) 51.52% and the second dominant signature in (1 out of 33) 3.03% of tumors. The average contribution value of HRd (SBS3) was 0.54 (range: 0.31–0.76). On the other hand, the HRd (SBS3) signature was detected in (4 out of 69) 5.8% of Non-HRd_TNBC tumors. HRd (SBS3) was the first dominant signature in (0 out of 69) 0% and the second dominant signature in (4 out of 69) 5.8% of such tumors. The average contribution value of HRd was 0.39 (range: 0.33–0.45). The SBS3 HRd (SBS3) signature presented higher prevalence and contribution in HRd_TNBC tumors than non-HRd_TNBC tumors (Figure 4).

An SBS39 signature was detected in (14 out of 33) 42.42% of HRd_TNBC tumors. SBS39 was the first dominant signature in (4 out of 33) 12.12% and the second dominant signature in (4 out of 33) 12.12% of tumors. The average contribution value of SBS39 was 0.32 (range: 0.25–0.4). In contrast, an SBS39 signature was detected in (3 out of 69) 4.35% of non-HRd_TNBC tumors. SBS39 was the first dominant signature in (0 out of 69) 0% and the second dominant signature in (3 out of 69) 4.35% of such tumors. The average contribution value of SBS39 was 0.27 (range: 0.23–0.32). In sum, the SBS39 signature presented higher prevalence and contribution in HRd_TNBC tumors than non-HRd_TNBC tumors (Figure 4).

An SBS8 signature was detected in (22 out of 33) 66.67% of HRd_TNBC tumors. SBS8 was the first dominant signature in (1 out of 33) 3.03% and the second dominant signature in (7 out of 33) 21.21% of the tumors. The average contribution value of SBS8 was 0.26 (range: 0.16–0.49). In contrast, an SBS8 signature was detected in (23 out of 69) 33.33% of non-HRd_TNBC tumors. SBS8 was the first dominant signature in none of them and the second dominant signature in (21 out of 69) 30.43% of such tumors. The average contribution value of SBS8 was 0.24 (range: 0.18–0.31). In sum, the SBS8 signature presented higher prevalence but lower contribution in HRd_TNBC tumors than non-HRd_TNBC tumors (Figure 4).

An NHEJd (ID8) signature was detected in (32 out of 33) 96.97% of HRd_TNBC tumors. NHEJd (ID8) was the first dominant signature in (14 out of 33) 42.42% and the second dominant signature in (17 out of 33) 51.52% of tumors. The average contribution value of NHEJd was 0.37 (range: 0.11–0.57). In contrast, an NHEJd (ID8) signature was detected in 30 out of 69 (90.91%) of non-HRd_TNBC tumors. NHEJd (ID8) was the first dominant signature in (0 out of 69) 0% and the second dominant signature in (15 out of 69) 21.74% of such tumors, and the average contribution value of NHEJd (ID8) was 0.25 (range: 0.15–0.39). In sum, NHEJd (ID8) presented higher prevalence and contribution in HRd_TNBC tumors than non-HRd_TNBC tumors (Figure 4).

An HRd (ID6) signature was detected in (33 out of 33) 100% of HRd_TNBC tumors. HRd (ID6) was the first dominant signature in (19 out of 33) 57.58% and the second dominant signature in (14 out of 33) 42.42% of tumors. The average contribution value of HRd (ID6) was 0.53 (range: 0.22–0.75). In contrast, an HRd (ID6) signature was detected in (4 out of 69) 12.12% of non-HRd_TNBC tumors. HRd (ID6) was neither the first nor the second dominant signature in any non-HRd_TNBC tumors, and the average contribution value of HRd (ID6) was 0.25 (range: 0.17–0.32). In sum, HRd (ID6) presented higher prevalence and contribution in HRd_TNBC tumors than non-HRd_TNBC tumors (Figure 4).

Notably, an MMRd signature was not detected in any HRd_TNBC tumors. However, an MMRd signature was detected in (2 out of 69) 2.9% of non-HRd_TNBC tumors. MMRd was the first dominant signature in (2 out of 69) 2.9% and the second dominant signature in (0 out of 69) 0% of tumors. The average contribution value of MMRd was 0.7 (range: 0.64–0.77) (Figure 4).

While an ROS signature was detected in none of HRd_TNBC tumors, it was detected in (15 out of 69) 21.74% of non-HRd_TNBC tumors. ROS was the first dominant signature in (0 out of 69) 0% and the second dominant signature in (12 out of 69) 17.39% of tumors. The average contribution value of MMRd was 0.19 (range: 0.11–0.3) (Figure 4).

We compared mutational signature profiles, the first and second dominant signature and the signature group of HRd_TNBC tumors to those of non-HRd_TNBC tumors. Enrichment of signatures in each tumor group were significantly different (first SBS dominant signatures: 2.7 × 10^−12^, second SBS dominant signatures: 1.5 × 10^−4^, SBS signature groups: 3.4 × 10^−9^, first ID dominant signatures: <2.2 × 10^−16^, second ID dominant signatures: 2.6 × 10^−11^, ID signature groups: 2.2 × 10^−16^ [*p* values; Fisher’s exact test]) (Figure 4c).

**Figure 5:**
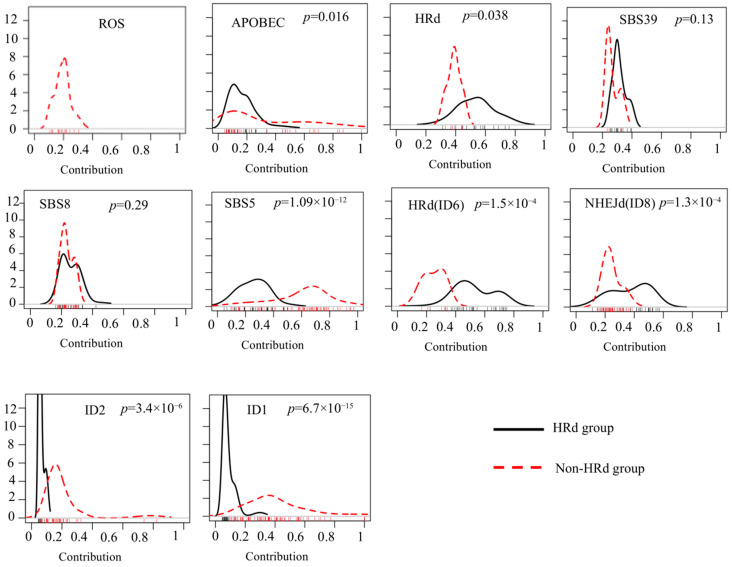
Significant difference in distribution of mutational signatures’ contribution in HRd and non-HRD TNBC tumors. TNBC cases were assigned to two distinct groups of HRd and non-HRd. The HRd group contain tumors with bi-allelic mutations of BRCA1/2 or PALB2, while the non-HRd TNBC group contains tumors with an HRd score less than 0.1. Distribution of mutational signatures’ contribution is compared using kernel density plots. The *X* axis represents contribution. Ticks for each contribution value are presented under the distribution curves. The *Y* axis is a kernel density estimate to show the probability density. Only samples with counts more than zero are shown. *p* values were calculated by the K.S. test.

We observed significant differences between the distribution shapes of SBS and ID signatures in HRd_TNBC tumors compared to non-HRd_TNBC tumors, except for SBS39 and SBS8, as shown in kernel density plots in Figure 5. Kernel plots further depict the extent and magnitude of difference between signature groups. ROS was not detected in HRd_TNBC tumors. However, it showed a shift towards lower contribution values with very narrow spread in non-HRd_TNBC tumors. In both HRd_TNBC tumors and non-HRd_TNBC tumors, large numbers of samples with APOBEC had a tendency towards the low contributions, while it was only in non-HRd_TNBC tumors that a subset of cases with higher contributions generated a quite long tail in the plot. SBS5 displayed significantly different distribution in HRd_TNBC tumors compared to non-HRd_TNB tumors. NHEJd (ID8) showed distributions towards higher contributions in HRd_TNB tumors compared to non-HRd_TNBC tumors. ID1 and ID2 showed narrower spread towards lower contributions in HRd_TNBC tumors compared with non-HRd_TNBC tumors (Figure 5).

## 4. Discussion

Analyzing 1014 breast cancer genomes, we identified distinct patterns of interaction between mutational signatures in different breast cancer types, which suggest possible clinical implications and precision therapeutic targets. The comprehensive profile of mutational signatures and their interactions revealed HRd and NHEJd have mutual exclusivity with ROS and affect APOBEC and aging mutational signatures in breast tumors. Notably, our findings revealed mutual exclusivity of MMRd and HRd mutational signatures in breast tumors, which is consistent with previous reports on gynecological [14], colorectal and stomach malignancies [15].

Two etiologically unknown signatures of SBS8 and SBS39 co-occurred with each other and were further enriched in higher-grade tumors. SBS8 and SBS39, while having high HRd scores, both showed mutual exclusivity with HRd (SBS3) signatures, indicating that cells with an HRd (SBS3) signature cannot tolerate additional deficiencies caused by SBS8 and SBS39. Notably, they both co-occurred with NHEJd (ID8) and HRd (ID6). The data suggest a possible role of these two signatures in DDR pathways associated with double-strand breaks and a highly likely HR pathway.

ROS induce significant damages to various targets including lipids, proteins and nucleic acids. These damages directly or indirectly exert detrimental effects during the multistage process of carcinogenesis, from initiation to malignant progression [47]. Although oxidative stress is often considered an adverse event, elevated levels of ROS can be toxic to cells, making them more vulnerable to further damage caused by exogenous agents. In fact, this characteristic of cancer cells can be employed for therapeutic benefits [48,49].

Based on our data, the ROS mutation signature (SBS18) shows the opposite behavior of SBS8 and SBS39. The ROS signature was mutually exclusive with HRd, APOBEC, SBS8 and SBS39. This suggests a possible notion for using ROS-related therapies in combination with HRd-related therapies to avoid emerging resistant tumor cells. Indeed, several studies reported context-dependent outcomes of ROS modulator combinations with chemotherapy and radiotherapy [26,27]. Therefore, the knowledge on the co-occurrence and exclusivity of ROS with other signatures may reflect the effect of ROS in the DNA damage response and its clinical application. ROS and oxidative distress are known to play a role in the induction and progression of breast cancer. On the contrary, highly elevated ROS levels may prevent tumorigenesis [27,50]. Understanding ROS regulation is highly significant since ROS modulation influences the outcome of common cancer therapies such as radio and chemotherapy drugs [51].

Notably, our findings on significant differential prevalence of ROS signatures among non-HRd TNBC tumors vs. HRd TNBC tumors (*p* value 0.0057, Fisher exact test) suggest a possible opportunity for therapeutic intervention. The lack of ROS among non-HRd TNBC tumors indicates that these cancer cells cannot tolerate the induction of double-strand breaks by HRd, while HRd tumors cannot tolerate ROS insults. Hence, it might be possible to preferentially eliminate these cancer cells by pharmacological interventions which induce HRd in the former and ROS insults in the latter.

NHEJd (ID8) co-occurred with APOBEC, HRd, SBS8 and SBS39, while it was mutually exclusive with MMRd and ROS. NHEJ is considered both a guardian and a disruptor of the genome, depending on the context of other genomic abnormalities [8]. In fact, a DNA repair crisis switch was reported to put pressure on NHEJ and drive chemotherapy resistance in TNBC tumors [52]. Notably, the INDEL signatures ID1, ID2 and ID4 were almost always mutually excluded from HR and NHEJ defects, perhaps suggesting (at least for ID1 and2) that replication fork slippage and subsequent DNA damage are dependent on rescue via HR/NHEJ.

HRd is defined from harboring damages of HR pathway-related genes to complex genomic scars [53]. Evaluating HRd status starts with performing germline testing of bi-allelic BRCA1/2 inactivation and then comprehensive approaches, such as measuring HRD scores and mutational signatures [54]. Measuring HRD scores relies on three independent factors, loss of heterozygosity (LOH), large-scale state transitions (LSTs) and telomeric allelic imbalance (TAI), while mutational signatures indicate the presence of HRd-associated mutation patterns across the entire genome. The importance of accurate and reliable detection of HRd status as an actionable maker underscores the requirement for integrating effective measurement approaches [55].

We showed that a relatively large proportion of tumors harbored HRd signatures manifesting a strong fingerprint of HRd such as high HRd scores and contribution values. The profile of mutational signatures in these tumors was dominated by HRd signatures, suggesting selective advantages of HRd signatures and their possible role in driving the mutational landscape of tumors and conferring aggressiveness. The results implicate potential benefits of detecting HRd signatures as actionable markers to guide identifying tumors sensitive to PARPi therapy as well as platinum-based chemotherapies. PARP plays a significant role in repairing single-strand breaks, especially via the BER pathway [56]. In HR-deficient cells, PARP inhibition results in simultaneous termination of two DDR pathways (HR and BER), which makes cells reliant on error-prone NHEJ for DNA repair, thus leading to the accumulation of DNA damage and ultimately cell death [57]. On the other hand, platinum treatment results in the accumulation of double-strand breaks. HRd tumors are fatally unable to repair double-strand breaks, thus enhancing their sensitivity to platinum agents. We also confirmed the presence of the MMRd signature in small subsets of breast tumors. These tumors dominated by the MMRd signature are potential candidates for immune checkpoint therapies. It has been suggested that sensitivity to checkpoint inhibitors in MMRd tumors is possibly due to excessive accumulation of genomic abnormalities that prime the immune system [58,59].

Genomic instability is a major contributor to cancer heterogeneity. Thus, reliably identifying patterns of genomic instability and turning it into an advantage for therapeutic targeting may result in improvements in the outcome of breast cancers. Cancer cells are extremely reliant on DNA damage/repair pathways, not only for their initial generation and but also subsequent survival. Therefore, personalizing the treatment of breast cancer patients whose tumors display specific DNA damage/repair-related defects according to their mutational signatures presents a promising approach for selecting targeted therapy. Our data collectively confirm that mutational signatures and their interaction faithfully reflect tumors’ defective DDR systems. Thus, we propose that the understanding of the mechanistic basis of mutational signatures and their etiology improves cancer diagnosis, prognosis and selection of therapy.

Conducting large clinical randomized trials with controlled monitoring of treatments and outcomes, in combination with comprehensive tumor genomics profiling, will clarify the clinical relevance of mutational signatures as markers for treatment selection and response and patient outcomes. These important questions remain to be addressed in future studies.

## 5. Conclusions

Herein, mutational signature analyses were employed to find favorable and unfavorable combinations via their interaction analyses. It provided the opportunity to identify promising synthetic lethal candidates for precision targeting of DNA repair systems. The presented data, which include the presence, prevalence and inter-relationship of mutational signatures in 1014 breast cancer genomes, provide a detailed and comprehensive resource for patient stratification and finding possible therapeutic targets. Considering the lack of the ROS signature in HRd TNBC tumors and its unique presence in non-HRd TNBC tumors, and also having an inverse relationship with HRd and NHEJd, the ROS signature can be one of those interesting candidates. The co-occurrence of HRd and NHEJd with APOBEC mutational signatures can be exploited to explore possible combination therapies as well. Moreover, interaction analysis suggests two etiologically unknown signatures of SBS8 and SBS39, which co-occurred with each other, may have a role in DDR pathways and are possibly associated with double-strand breaks.

## Data Availability

All data generated or analyzed during this study are included in this published article and its Supplementary Information files. All R packages and scripts used for the analyses are available publicly as described in the Methods section. The ICGC dataset is available at https://dcc.icgc.org/projects, accessed on 7 May 2025.

## Supplementary Materials

The following supporting information can be downloaded at: https://www.mdpi.com/article/10.3390/cancers17101627/s1, Supplementary Figure S1: Mutational signature analysis of breast tumors genome. We analyzed SBSs, IDs, and DBSs in 777 whole genomes of breast tumors. (a) Non-Negative Matrix Factorization (NMF)-based de novo mutational signatures of tumors visualized by a heatmap divided based on distinct signature status. Signature group, the first and second dominant SBS and ID signatures, and tumor grades, are annotated at the top. The contribution values of each signature are shown by a color scale. Color codes representing each dominant mutational signature are shown. For mutational signatures with known etiology, both signature and etiology are indicated. (b) Interaction of signatures with each other measured by hypergeometric test: red represents co-occurrence and blue represents mutual exclusivity. Supplementary Figure S2: TMB of SBS, ID and DBS signatures for breast and TNBC tumors. (a) TMB of 777 breast tumors. (b) TMB of 237 TNBC tumors. TMB is measured in somatic mutations per Megabase (Mb). In the TMB plots, columns represent the detected mutational signatures and are ordered by mean somatic mutations per Mb from the lowest frequency, left, to the highest frequency, right. Numbers at the bottom of the TMB plots represent the numbers of tumors harboring each mutational signature. Only samples with counts more than zero are shown. Supplementary Figure S3: Dominant signature analysis of breast tumors genome. (a) The first and second dominant SBS mutational signatures. (b) The first and second dominant ID mutational signatures. Dominant signatures were based on the contribution value of detected mutational signatures. For mutational signatures with known etiology, both signature and etiology are indicated. Supplementary Figure S4: Dominant signature analysis of TNBC tumors genome. (a) The first and second dominant SBS mutational signatures. (b) The first and second dominant ID mutational signatures. Dominant signatures were based on the contribution value of detected mutational signatures. For mutational signatures with known etiology, both signature and etiology are indicated.
