# Supplementary material for: The Use of Mutational Signatures to Decipher the Inter-Relationship of Reactive Oxygen Species with Homologous Recombination and Non-Homologous End-Joining Deficiencies as Well as Their Effects on APOBEC Mutagenesis in Breast Cancer
Source: Cancers (Basel). 2025 May 12;17(10):1627. doi: 10.3390/cancers17101627 (PMC12110613; doi:10.3390/cancers17101627)

Mutational signatures decipher inter-relationship of reactive oxygen species with homologous recombination and non-homologous end-joining deficiencies, their effects on APOBEC mutagenesis in breast cancer

Supplementary Figures

Supplementary Figure S1. Mutational signature analysis of breast tumors genome. We analyzed SBSs, IDs, and DBSs in 777 whole genomes of breast tumors. (**a**) Non-Negative Matrix Factorization (NMF)-based *de novo* mutational signatures of tumors visualized by a heatmap divided based on distinct signature status. Signature group, the first and second dominant SBS and ID signatures, and tumor grades, are annotated at the top. The contribution values of each signature are shown by a color scale. Color codes representing each dominant mutational signature are shown. For mutational signatures with known etiology, both signature and etiology are indicated. (**b**) Interaction of signatures with each other measured by hypergeometric test: red represents co-occurrence and blue represents mutual exclusivity.

Supplementary Figure S2. TMB of SBS, ID and DBS signatures for breast and TNBC tumors. (**a**) TMB of 777 breast tumors. (**b**) TMB of 237 TNBC tumors. TMB is measured in somatic mutations per Megabase (Mb). In the TMB plots, columns represent the detected mutational signatures and are ordered by mean somatic mutations per Mb from the lowest frequency, left, to the highest frequency, right. Numbers at the bottom of the TMB plots represent the numbers of tumors harboring each mutational signature. Only samples with counts more than zero are shown.

Supplementary Figure S3. Dominant signature analysis of breast tumors genome. (**a**) The first and second dominant SBS mutational signatures. (**b**) The first and second dominant ID mutational signatures. Dominant signatures were based on the contribution value of detected mutational signatures. For mutational signatures with known etiology, both signature and etiology are indicated.

Supplementary Figure S4. Dominant signature analysis of TNBC tumors genome. (**a**) The first and second dominant SBS mutational signatures. (**b**) The first and second dominant ID mutational signatures. Dominant signatures were based on the contribution value of detected mutational signatures. For mutational signatures with known etiology, both signature and etiology are indicated.

Supplementary Figure S1

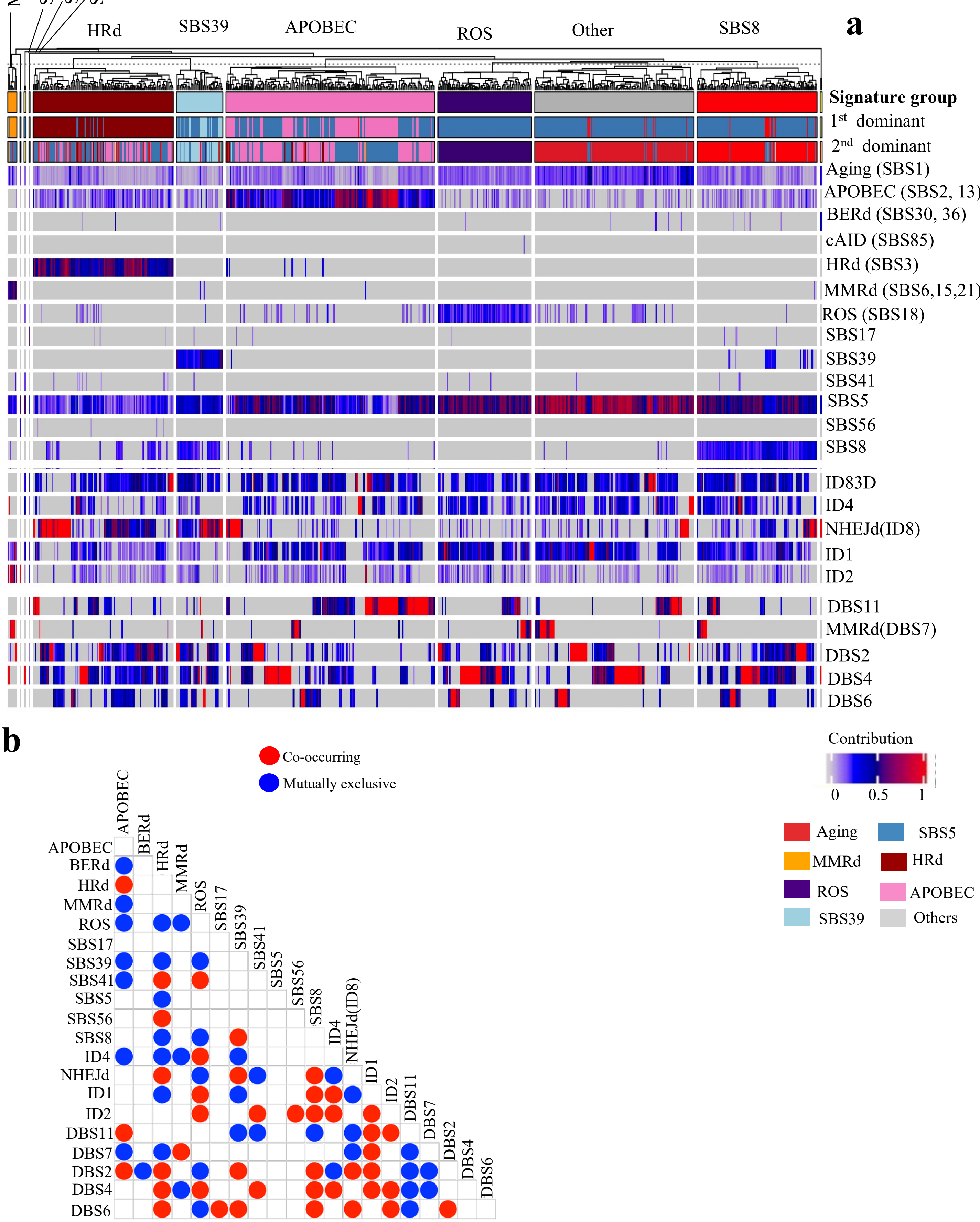

Supplementary Figure S2

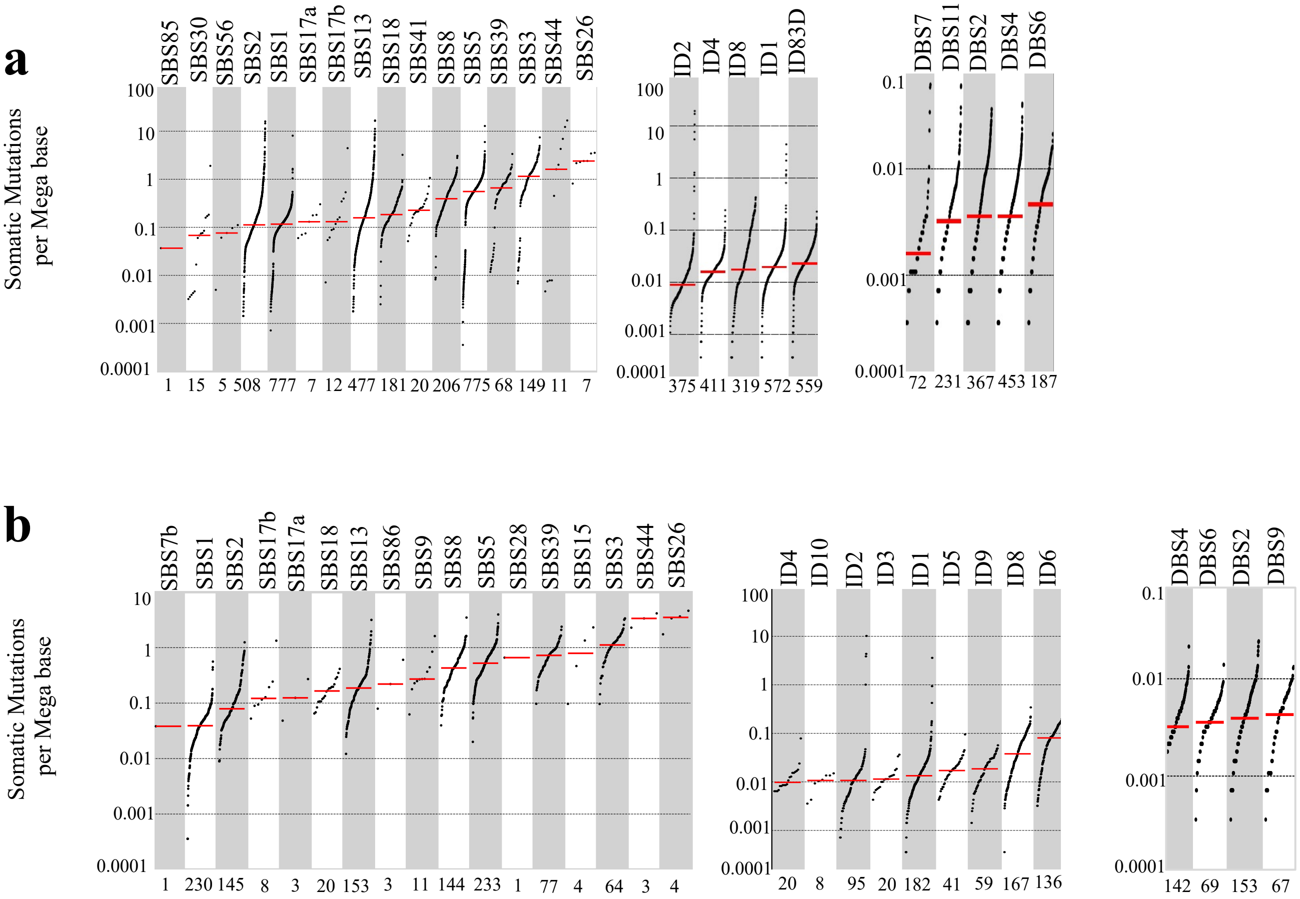

Supplementary Figure S3

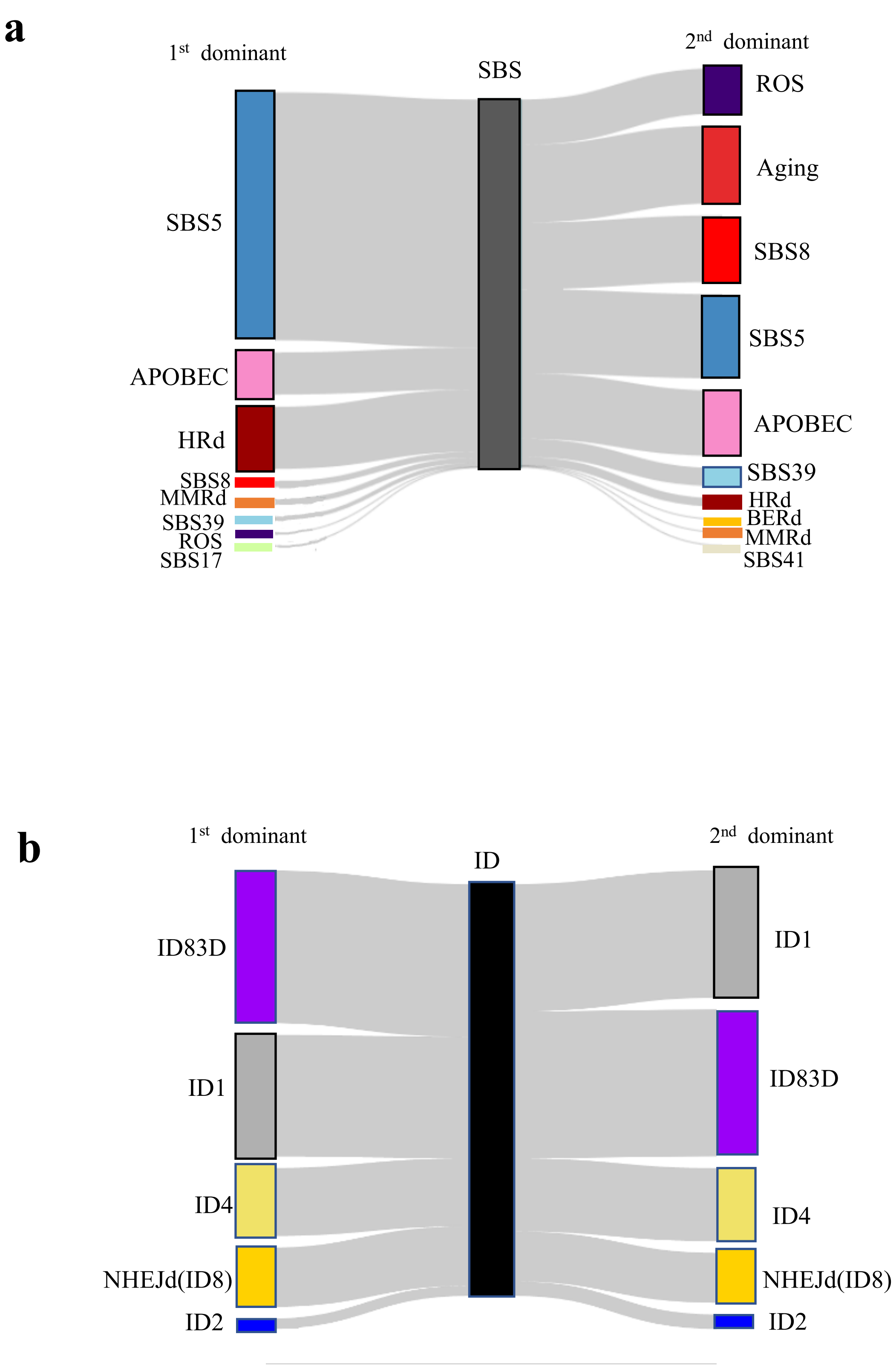

Supplementary Figure S4

a

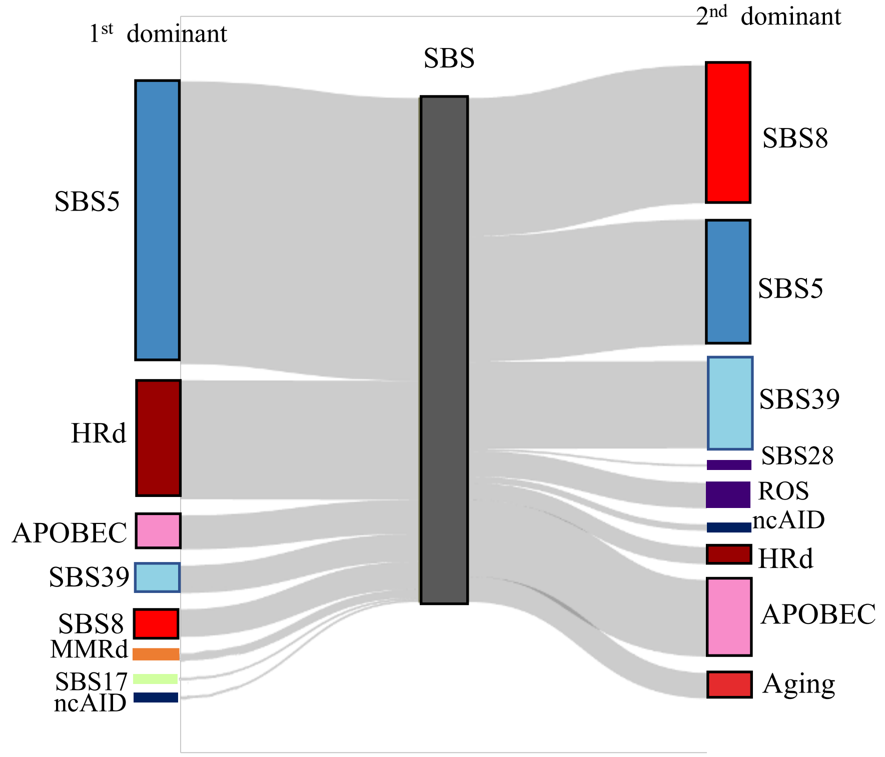

b

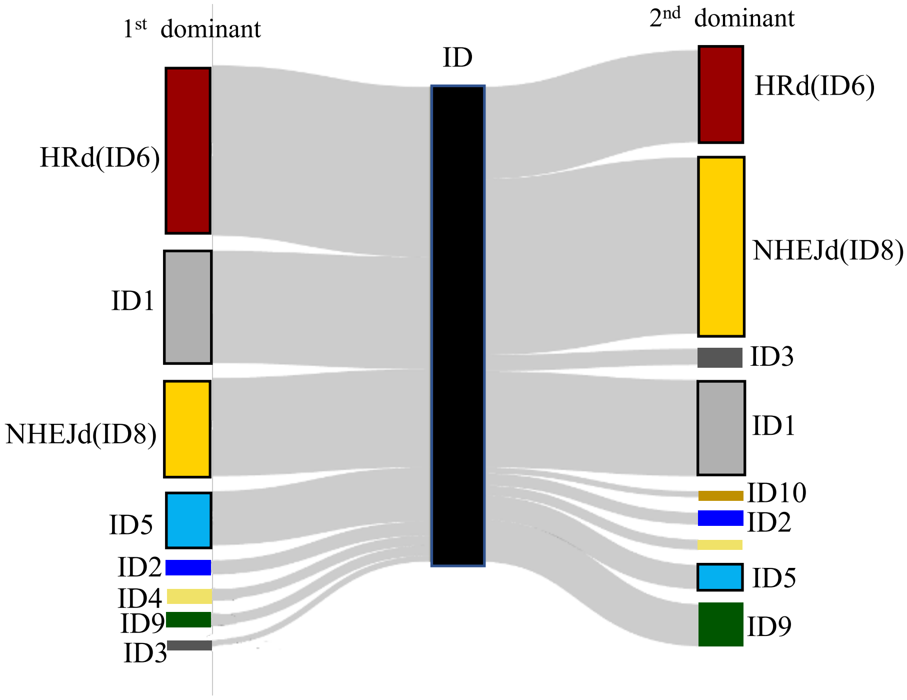

Supplement: Supplementary file 1 [file cancers-17-01627-s001.zip › cancers-3597270-supplementary.pdf]
